# External Auditory Stimulation as a Non-Pharmacological Sleep Aid

**DOI:** 10.3390/s22031264

**Published:** 2022-02-07

**Authors:** Heenam Yoon, Hyun Jae Baek

**Affiliations:** 1 Department of Human-Centered Artificial Intelligence, Sangmyung University, Seoul 03016, Korea; h-yoon@smu.ac.kr; 2 Department of Medical and Mechatronics Engineering, Soonchunhyung University, Asan 31538, Korea

**Keywords:** sleep, auditory stimulation sleep induction, digital therapeutics, sleep aid

## Abstract

The increased demand for well-being has fueled interest in sleep. Research in technology for monitoring sleep ranges from sleep efficiency and sleep stage analysis to sleep disorder detection, centering on wearable devices such as fitness bands, and some techniques have been commercialized and are available to consumers. Recently, as interest in digital therapeutics has increased, the field of sleep engineering demands a technology that helps people obtain quality sleep that goes beyond the level of monitoring. In particular, interest in sleep aids for people with or without insomnia but who cannot fall asleep easily at night is increasing. In this review, we discuss experiments that have tested the sleep-inducing effects of various auditory stimuli currently used for sleep-inducing purposes. The auditory stimulations were divided into (1) colored noises such as white noise and pink noise, (2) autonomous sensory meridian response sounds such as natural sounds such as rain and firewood burning, sounds of whispers, or rubbing various objects with a brush, and (3) classical music or a preferred type of music. For now, the current clinical method of receiving drugs or cognitive behavioral therapy to induce sleep is expected to dominate. However, it is anticipated that devices or applications with proven ability to induce sleep clinically will begin to appear outside the hospital environment in everyday life.

## 1. Introduction

Quality nocturnal sleep is related to a variety of quality performances in our lives, including maintaining health and improving outcomes at work. On the other hand, poor-quality sleep causes not only daytime fatigue and sleepiness, but also emotional issues and can even induce sicknesses such as heart disease, hypertension, anxiety, and depression [1]. Short sleep onset latency is one of the most important aspects of good sleep. A sleep onset latency less than 30 min is considered a good indicator of sleep and the most defensible quantitative factor in clinical practice [2]. In clinical trials using pharmacological [3,4] or non-pharmacological treatment for insomnia [5,6], sleep latency less than 30 min was an indicator of effectiveness. In the past, insomnia was not regarded as an independent disease but as a secondary symptom of other disorders and insomnia was classified into secondary insomnia and primary insomnia. Secondary insomnia refers to that caused by other medical/psychiatric disorders or substances/drugs; that without such a cause is called primary insomnia. Secondary insomnia accounts for 70–90% of insomnia, although it is not easy to differentiate primary insomnia and secondary insomnia in clinical practice. Secondary insomnia must occur with or immediately after onset of the cause, and when the cause disappears, the insomnia must also disappear. However, it became apparent that this was not always the case, and it was argued that independent insomnia can exist even if a disease was suspected to be the cause [7,8]. Insomnia is a prevalent sleep problem, even in the general population without a specific medical condition. In the United States, the annual prevalence of insomnia symptoms and insomnia disorders has been reported to be between 35–50% [9] and 10–22% [10,11], respectively. About one-third of the European population is reported to have experienced one or more symptoms associated with insomnia, such as difficulty in initiating or maintaining sleep [12,13]. The prevalence of insomnia symptoms and diagnosis in Korea was reported to be 17–23% and 5%, respectively [14,15]. In addition, the National Health Insurance Service-National Sample Cohort (NHIS-NSC) from 2002–2013 showed that the annual incidence of insomnia over recent years remained steady, but the prevalence increased for the general population in Korea [16]. In particular, studies worldwide have reported that the incidence or risk of insomnia has increased significantly due to various physical and psychological problems during the COVID-19 pandemic [17,18,19,20].

In the era of the Internet of Things, medical practices are connected with smartphones and wearable devices, opening an era of ubiquitous, mobile, and digital healthcare. These healthcare devices can be used for self-monitoring or self-evaluation to help individuals better understand their behavior, body, and health and can be tracked and analyzed to modify individual behaviors. Most wearable devices that monitor sleep use actigraphy, which is based on an accelerometer worn on the wrist to detect movement of the limbs and distinguish whether the wearer is sleeping or awake. Although this has the advantage of being able to be used repeatedly at home for several nights, compared to polysomnography performed at the hospital or sleep laboratory, it does not detect drowsiness, overestimates the time it takes to fall asleep, and underestimates the number of awakenings during sleep [21]. Actigraphy now can measure the heart rate and identify sleep stages based on heart rate variability due to sympathetic or parasympathetic modulation of the autonomic nervous system. Monitoring of sleep quality, sleep stages, and sleep disorders such as apnea is being studied continuously, and some devices have been commercialized and used by consumers, although the technology to solve the detected sleep problems is insufficient. Technology that solves and even treats health-related problems such as sleep disease is a trend in digital therapeutics (DTx). DTx uses digital technology and medical care combined with the advantage of significantly lower time and cost for development compared to existing treatments. Therefore, it is attracting attention as a third-generation therapeutic agent following the first-generation therapeutic low-molecular-weight compounds (pills or capsules) and second-generation therapeutic biological products (antibodies, proteins, and cells). For patients with clinically diagnosed insomnia, cognitive behavioral therapy is recommended as the first treatment. Cognitive behavior therapy consists of stimulation control, sleep restriction, relaxation therapy, paradoxical intentions, and sleep hygiene education. Stimulation control prohibits subjects from doing anything other than sleeping in bed, and sleep restriction reduces the amount of time subjects spend in bed and prevents subjects from resting unnecessarily. Relaxation therapy is a training technique that lowers tension through relaxation of muscles or breathing. Paradoxical intention is a method of deliberately trying not to sleep. Sleep hygiene education is focused on the environment or habits that help or interfere with sleep. Stimulation control, sleep restriction, relaxation therapy, and paradoxical intentions can be recommended alone or as a treatment for chronic insomnia, but cognitive behavioral therapy often is performed by integrating the above techniques [22,23]. Digital application of cognitive behavioral therapy delivered using web services and even mobile apps has shown promise in the treatment of insomnia [24,25]. As such, digital cognitive behavioral therapy is effective as DTx to treat sleep problems (insomnia), but there are limitations. If cognitive behavioral therapy is performed using digital technology such as a smartphone, it is difficult to personalize, and the dropout rate is high due to lack of encouragement from a therapist. In addition, if the user does not feel that the insomnia has improved, the will to participate in treatment is greatly reduced [26,27].

In the past, it was believed that a quiet environment was important for quality sleep. Noise, previously defined as unintentional sounds during sleep, can have negative psychological and physical effects, including cardiovascular stimulation, hearing loss, increased gastric secretion, stimulation of the pituitary and adrenal glands, suppression of immune responses to infections, and female fertility [28,29,30,31]. However, noise, which was once avoided for quality sleep, has been adopted recently by the general public as a non-pharmacological sleep inducer. In an online survey of the general population, 403 of 651 participants (62%) said they had used sound sources at least once to help them sleep [32]. Additionally, in a survey of more than 500 sleep-disordered patients, more than 50% reported using a sound source as a sleep aid [33]. Some products that use auditory stimulation as a sleep promoter have been commercialized. Table 1 summarizes the currently available sleep promotion devices or smartphone apps that use auditory stimulation. The teardrop-shaped Sound + Sleep Mini allows users to select from 48 sounds to induce sleep, including rain, stream, fan, sea, and color noises. Lectrofan produces 18 sounds, half of which are from various fans and vents. The other half are colored noises such as white, pink, or brown noise. Other products feature similar soundscapes. While most devices are sound machines that provide different auditory stimuli to induce sleep, Hatch Restore offers additional features such as color-changing lights, a digital clock display, and a mobile app. Hatch Restore also has a selection of relaxing music and narration in addition to the sounds of rain, waterfalls, whales, and other relaxing sounds. In addition, many paid or free apps provide these types of sounds through smartphones. Table 1 summarizes two representative apps that use different sound sources from previous sleep-inducing devices. Sleepmaker Rain1 is an app that induces sleep using the sound of rain as an auditory stimulus and is characterized by various types of rain sounds, such as the sound of rain in the forest, the sound of rain and wind hitting a window, the sound of rain falling on the roof, and the sound of heavy rain. The user can select the auditory stimulation time from 1 min to 23 h 59 min. The sleep app features the ability to choose from a variety of natural sounds, city sounds, colored noises, or instrument sounds and to mix them to create personalized auditory stimuli.

As shown in Table 1, many sleep-related devices, as well as smartphone apps, use auditory stimulation to induce sleep, but the effect has not been validated. This review provides the current level of non-pharmacological sleep induction methods, including investigation of auditory stimuli for sleep induction and experimental validation results. The review methodology consists of collecting as many series of processes from the literature as possible using keywords and then reviewing the selected literature in detail according to a set standard. First, keywords of auditory stimuli were defined to obtain detailed information about the various sound sources used in sleep studies and to collect results. Then, the topics of various studies searched for sleep and keyword sound sources were assessed, excessive content was omitted, and relevance with defined keywords was determined. Next, we reviewed the abstracts and determined whether the research papers were related to sleep induction. Search selection was limited to literature containing experimental results evaluating sleep latency, and studies without evaluation were removed. Selected papers and citations were evaluated. Finally, full collected papers were reviewed comprehensively. Accordingly, the types of sound sources used for sleep induction were divided into three categories: (1) pure colored noise, (2) autonomous sensory meridian response (ASMR), and (3) music. In each category, eight studies used pure colored noise, three studies used ASMR, and eight studies used music. For a total of 19 studies, detailed types of sound sources, experimental methods, and sleep test results were reviewed.

## 2. Pure Noise

**Figure 1:**
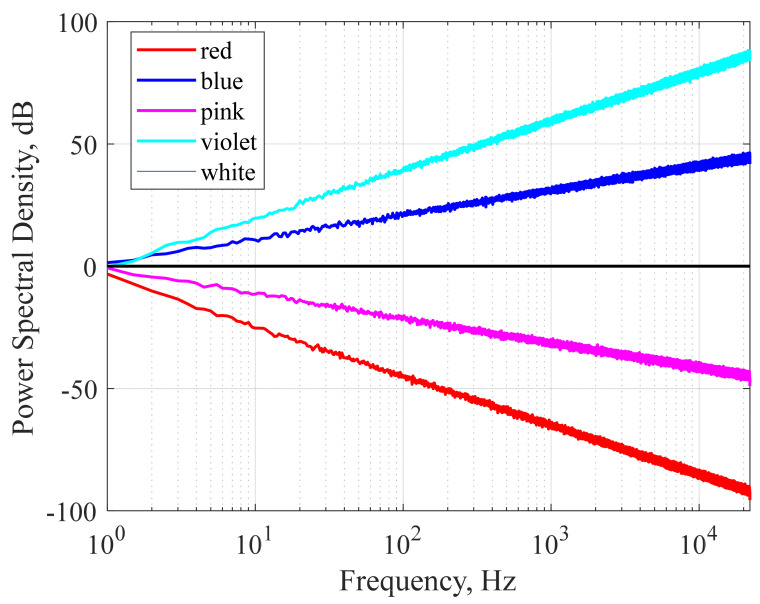
Simulated power spectral density as a function of frequency for different colored noise (red, blue, pink, violet, and white). The power spectral density is normalized arbitrarily so that the spectral values are approximately 1 Hz.

**Figure 2:**
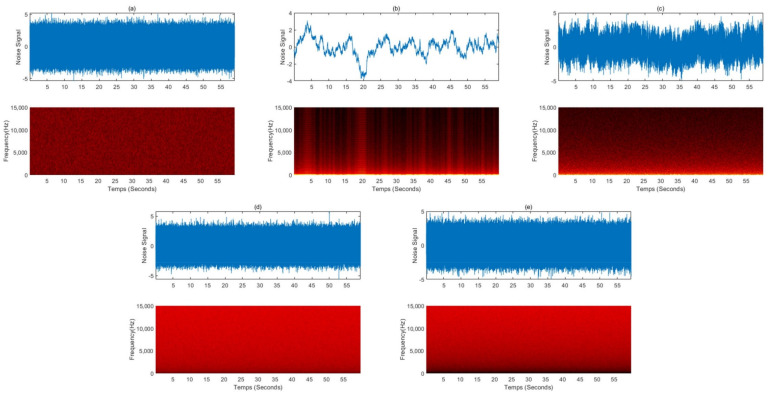
Simulated colored noise signals and spectrograms: (**a**) white noise, (**b**) red noise, (**c**) pink noise, (**d**) blue noise, and (**e**) violet noise.

The characteristics of sound waves are described mainly by frequency and amplitude. Frequency refers to how fast a wave vibrates per second, and amplitude refers to the size of the wave. The relationship between the frequency and amplitude of a sound wave is used to define colors of noises that share structural properties with the corresponding light wave of the same name. One of the most famous applications of auditory stimulation to induce sleep is white noise. The term white noise is often used to describe a general hissing sound, which is mostly pure noise without meaning. However, various sounds that are labeled white noise can be expressed as colored noises such as white, pink, and brown according to characteristics in the power spectrum. Figure 1 showed simulated power spectral densities as a function of frequency for various colored noise. We simulated colored noise, and the spectrograms are shown in Figure 2.

The studies examining the sleep-inducing effect of pure colored noise are summarized in Table 2. White noise (Figure 1 black line) is a signal that has equal power in any band of the frequency spectrum of a given bandwidth. Since white noise contains all frequencies with equal intensity, it can mask other external noises, eliminating arousal stimuli and calming the user. A study involving 2 groups of 20 neonates in a randomized trial by Spencer et al. found that 16 (80%) babies fell asleep within 5 min in response to white noise, while only 5 (25%) in the control group fell asleep spontaneously [34]. In a study by Forquer and Johnson, four college students were given a white noise generator and instructed to use it overnight for one week. All of these students showed a decrease in sleep latency and nocturnal arousal during the week of exposure. However, at one month of follow-up after cessation of exposure to white noise, these sleep-improving effects were lost. They reported that white noise-induced sleep improvement was only effective when administered actively, in a similar way to drugs such as sleeping pills [35]. Ebben et al. reported that white noise significantly reduced sleep latency and wake after sleep onset (WASO) based on subjective and objective measurements (sleep diary) of 10 adult participants living in a high-noise environment in New York City [36]. Cho et al. investigated the effects of white noise on sleep in hospitalized patients in a randomized controlled trial design. The experimental group (*n* = 30) listened to white noise for 1 h before sleeping, and the control group (*n* = 31) wore earplugs before sleeping. The white noise group showed a positive effect on subjective sleep quality compared to the earplug group and reported that the sleep efficiency measured by the wearable device improved significantly [37]. Messineo et al. studied the sleep-inducing effect of white noise in 18 healthy subjects with two nocturnal sleep studies approximately one week apart. They were exposed randomly to either normal environmental noise or white noise uniformly delivered by two speakers in a room. To model transient insomnia, subjects went to bed 90 min before their usual bedtime. Experimental results showed that white noise significantly reduced sleep onset latency by 38% compared to normal environmental noise [38]. Pink noise, also called 1⁄f noise, is a signal with a frequency spectrum whose power spectral density is inversely proportional to the frequency of the signal. Like white noise, pink noise is known to induce good-quality sleep by masking surrounding arousal stimuli. However, pink noise can be softer and more soothing as it uses a deeper sound and lower sound waves than white noise. Because the human ear is particularly sensitive to high frequencies, the general public finds pink noise more pleasant than white noise. Pink noise became popular because many researchers chose to induce brain waves that appear during deep sleep or stage three slow waves, the time when the body performs most long-term memory integration [39,40]. However, in addition to studies to induce deep sleep or improve memory, some claim to have potential as sleep-inducing agents. Kawada et al. studied the effect of pink noise as a sleep inducer in four young students in a repeated experiment. As a result of the experiment, the average sleep onset latency during sleep exposed to pink noise was 13.5 min compared to 23 min in the control experiment, a 58% improvement [41]. Garcia-Molina et al. studied the sleep-inducing effect by controlling the volume of pink noise using parameters derived from the power of beta and theta waves from a single EEG (FPz-M2). The sound was played through a wearable headband connected to the audio output of a laptop, and when the parameters calculated from the measured EEG met the requirements of the algorithm, the volume of the pink noise was reduced to zero. An experiment on seven healthy adult participants confirmed that the sleep latency decreased by an average of 2.2 min as a result of pink noise volume control through the proposed method [42]. Brown noise, also called red noise, is that with a power spectral density inversely proportional to f^2^, meaning it has higher intensity at lower frequencies, even more so than white and pink noise. It decreases in intensity by about 6 dB per octave, resulting in deeper intensity than pink and white noise and sounds like a low roar. In contrast with brown noise, violet noise, also called purple noise, increases at a rate of 6 dB per octave. Blue noise and violet noise have similar constants and low frequency, but blue noise is less biased toward higher frequencies. The power density of blue noise increases by 3.01 dB per octave with increasing frequency. Several prominent international media providers are drawn to the use of brown, purple, and blue noise as sound for inducing sleep because it is less disturbing than white noise. In addition, many sound sources with color noise such as brown, purple, and blue are uploaded to media such as YouTube under the title of Sleep Induction. However, scientific evidence is insufficient as there are few related academic reports.

## 3. Autonomous Sensory Meridian Response (ASMR)

**Figure 3:**
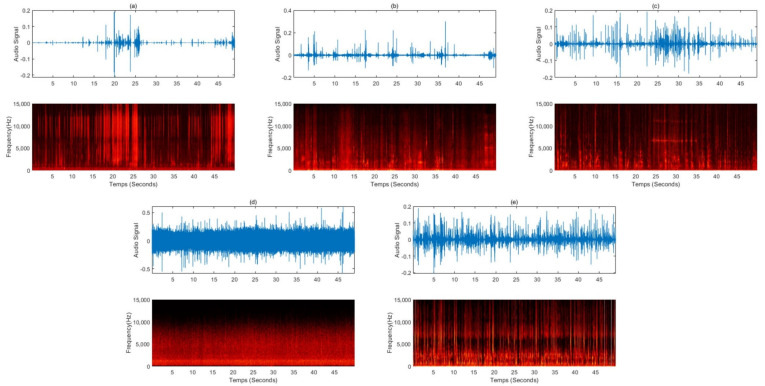
Sound waves and spectrograms for a 50-s sound source randomly extracted from videos uploaded to YouTube for sleep induction: (**a**) the sound of making glossy slime with a piping bag, (**b**) a voice whispering softly in a hair shop role play, (**c**) the sound of an oil massage and the whispering sound overlapped, (**d**) the sound of raindrops falling from the eaves and raindrops pouring, (**e**) steel wool sponges, the sound of rubbing various brushes, dolls, paper packaging materials, toys, etc., into the microphone.

In recent years, there has been growing interest in people who experience a previously unknown sensory phenomenon known as ASMR [43]. Many people use ASMR to achieve quality rest and use it as a means to treat depression, stress, and chronic pain. However, there are insufficient studies on ASMR. To date, there is no rigorous scientific definition of ASMR or the conditions that trigger or end an ASMR state. Therefore, a visual, auditory, or tactile stimulus that helps to relax and creates a sense of psychological well-being or pleasure is called ASMR. Early ASMR was limited to the sounds of clicking keyboards, cutting paper with scissors, and other simple everyday sounds. It now features natural ambient sounds such as rain and bustle of the city and has evolved to take advantage of sounds such as brushing hair, tapping, scratching, whispering, and role-playing sounds. Table 3 summarizes the representative five videos uploaded on YouTube in 2021 when searched with the keyword “sleep induction ASMR”. Figure 3 is a spectrogram analyzed by randomly collecting 50 s of data for an ASMR sound source with a length of about 10 to 60 min, as shown in Table 3. The ASMR sound source for inducing sleep showed features corresponding to white or pink noise intermittently over time when viewed in the frequency domain. In Figure 3d, which shows a similar rain sound from the beginning to the end of the sound source, the characteristics of pink noise are well shown. Therefore, it is possible to explain the sleep-inducing effect of the ASMR sound sources through pure noise.

Though various ASMRs for inducing sleep have become popular and received widespread media attention, they are little recognized by the scientific community. Only three studies have verified experimentally the sleep-inducing effect of ASMR (Table 4). Williamson investigated the effect of ocean sound on sleep before the term ASMR was coined [44]. In that study, sleep was assessed using the Richards–Campbell Sleep Questionnaire in 60 patients who underwent coronary artery bypass grafting (CABG) after being transferred from the intensive care unit. There was no significant difference in falling asleep score, but there were significant differences in sleep depth, awakening, return to sleep, quality of sleep, and total sleep scores before and after listening to ocean sound. The group that listened to the sound of the ocean reported higher scores indicating better sleep. A study by Hardian et al. appears to be the first to use the term ASMR in the effect of auditory stimulation on sleep [45]. They conducted a randomized controlled study of 30 people in which the ASMR intervention group watched a 20-min ASMR YouTube video at 9:00 pm for 14 consecutive days. The ASMR videos used in the experiment took the form of role-plays such as haircuts, cooking, etc., and also included whispers, rubbing, delicate hand movements, and elements that arouse meticulous personal attention. The experiment reported a significantly decreased Pittsburgh Sleep Quality Index (PSQI) score and improved quality of sleep, but details such as sleep latency were not reported. In a study by Umbas et al., a crossover design experiment was conducted with 12 people [46]. Subjects listened to rain sounds for one hour from 10:00 pm during the 30 days of ASMR intervention, and subjective sleep quality before and after ASMR intervention was assessed using the PSQI. The experiment showed significantly decreased PSQI score and increased quality of sleep. In particular, some subjects reported improvement in sleep latency. With the growing popularity of ASMR, various types of sleep-inducing tools are appearing on media such as YouTube, but research on the effect of ASMR on sleep is lagging. In a study by Williamson and Umbas [44,46], natural sounds such as rain and sea were used as ASMR stimulation. Most natural sounds, such as wind, rain, and water flow, belong to the white noise family.

## 4. Music

In general, it has been documented that the use of music as a health-related therapeutic tool is intended to activate or calm our inner state. For example, it uses activating rhythms and strong beats to activate our inner state. Several studies have reported that listening to music alleviates sleep disturbance problems, including insomnia. It has been reported that listening to music reduces heart rate, blood pressure, and respiration rate and even reduces activity of the sympathetic nervous system [47]. To induce sleep, we primarily use slow, repetitive rhythms, which create a sense of safety and security that can prepare and elicit a sleep response in the brain [48]. In this process of stabilizing vital signs, serum noradrenaline-reducing hormone is believed to promote a state of deep relaxation and calm, which aids in the onset of sleep [49]. Table 5 summarizes studies using music for sleep induction. An experiment performed by Iwaki et al. was conducted by dividing 20 college students into two groups [50]. The first group was instructed to sleep naturally when they wanted, and the second group was instructed to fall asleep as soon as possible. These two groups performed the listening to music experiment with a control condition of absence of music at intervals of 1 week. Experiments have shown that music promotes falling asleep in the natural sleep group (sleep whenever you want), whereas music interferes with falling asleep in the attempted sleep group (sleep as quickly as possible). Subjects were asked to listen to their preferred music, but information on what kind of music they listened to is not disclosed. Johnson et al. found that, in 52 women over 70 years of age with insomnia, the use of the subjects’ selected music at bedtime helped to increase the level of sleepiness, reduce sleep onset times, and reduce the number of nighttime awakenings [51]. In this experiment, participants were instructed to choose their own music to listen to at bedtime, and the choices could vary from night to night if they were in the same category such as classic music during 10 nights of sleep. The majority of participants chose soothing classical music such as Pachelbel’s Canon D or Bach at Bedtime, while the remainder chose sacred music or new age music. Lai et al. conducted a randomized controlled trial of a total of 60 people aged 60 to 83 years with sleep difficulties and found that classical music decreased the sleep latency [52]. Bloch et al. investigated the effect of musical relaxation on insomnia in people with schizophrenia [49]. Twenty-four people with schizophrenia were subjected to a 7-day treatment-free period followed by another 7-day study in which music was played at bedtime. The music used in the experiment consisted of diatonic notes of an arpeggio chord especially composed for the study and was performed using a piano and a violin. The experiment found that sleep latency and sleep efficiency were improved after playing relaxing music. Shum et al. recruited a cohort of 60 elderly people aged 55 or older in a randomized controlled study [53]. Experimental group participants were asked to listen to soft, slow, soothing instrumental music without lyrics for 40 min per day for 6 weeks. Experimental results showed that such instrumental music without lyrics shortened sleep latency. Wang et al. performed a randomized controlled study of 64 elderly people over the age of 60 with poor sleep quality with a PSQI higher than 7 [54]. The experimental group was asked to select and listen to preferred music from a music database for 30–45 min at night for 3 months. The music intervention group showed greater improvements in sleep latency and daytime dysfunction, sleep latency, sleep duration, and sleep efficiency. In general music tuning, the A (D) note is set to 440 Hz. This is the standard set by the International Organization for Standardization (ISO) in 1953, and most music follows this tuning. However, recently, in internet-based media, there have been claims that 432 Hz tuning can be beneficial physically, psychologically, and even spiritually. Against this background, Dubey et al. investigated the effect of 432 Hz music on sleep quality and sleep latency during a nap in 15 subjects with a history of delayed sleep latency [55]. All subjects underwent a sleep study at 1-week intervals, with or without 432 Hz music intervention. The results of the study showed a decrease in average sleep latency with a significant increase in alpha wave energy at the onset of sleep. Jesperson et al. performed a blinded, randomized, controlled study of 57 patients with insomnia assigned randomly to a music intervention group, an audiobook control group, and a waiting list control group [56]. The subjects in the music intervention group were allowed to choose among slow-tempo music of classical, jazz, new age, and ambient genres. Music intervention for at least 30 min at bedtime for 3 weeks each day had a significant effect on subjectively judged sleep improvement and quality of life, unlike the other control groups. In addition, although not statistically significant, sleep onset latency showed a tendency to decrease.

## 5. Discussion

### 5.1. Limitation

It is commonly believed that sleep can be induced through auditory stimulation such as colored noise, ASMR, and music, and various sleep-inducing products including white noise machines have emerged accordingly. In particular, various types of unverified sound sources are being released on various social media platforms such as YouTube under the name of sleep-inducing sound sources. After reviewing the published scientific literature, we concluded that there is evidence to support these claims, but the quality of the evidence is poor. A sleep diary is a tool with separate components that need to be filled out each day at bedtime and at waking. The bedtime component relates to the events the day before sleep, and the wake-up time component relates to the sleep duration just completed. Although it is the most widely used tool for subjective sleep evaluation, it has the disadvantage that it is not as accurate as an objective measure. In a study comparing sleep diary and polysomnography for depressed insomniacs, although there was a correlation in the sleep records recorded by the two methods, there was a statistically significant difference. In the sleep diary, sleep onset latency, latency to persistent sleep, and WASO were significantly longer than PSG, and total sleep time (TST) was significantly shorter [57]. This is consistent with other studies showing that a sleep diary overestimates TST compared to polysomnography [58,59]. Although a variety of sleep assessment methods exist, no method demonstrates complete agreement with actual sleep parameters, so researchers should carefully consider which sleep assessment method best matches the research question and parameters of interest. For an accurate evaluation of the sleep-inducing effect of auditory stimulation, the sleep assessment tool that can accurately measure parameters related to sleep time should be used, and sleep diaries are considered to have limitations in this respect. Among the studies reviewing the effects of auditory stimulation on improvement of sleep latency reviewed in this article, only about 31% (6 of 19 publications) presented objective results through polysomnography. In the remaining 69%, effectiveness was assessed using tools such as sleep diaries, records by observers, and sleep surveys. Therefore, to present a scientific basis for the sleep-inducing effect of auditory stimulation, a follow-up study including verification of polysomnography in parallel is necessary.

Interest in ASMR is increasing day by day, and while countless ASMR content is being generated, research on physiological and medical responses to ASMR is only in its infancy. As of the 2010s, research literature on ASMR has been published, but only three papers evaluating the sleep latency for ASMR stimulation were searched. Of these, two cases used continuous natural sounds, such as rain, and only in a study by Hardian et al. published in 2020, role-playing whispers, rubbing sounds, and cutting sounds were used, which are recently popular as ASMR sound sources. Therefore, as in the case of studies using colored noise or music, where relatively more studies have been reported, there is a need for a number of clearly designed studies using stimulation sources of various ASMR genres targeting groups of subjects from various cultures, various age groups, and various environments. According to the literature researching the reaction to ASMR, the genre of ASMR content that causes a neural reaction may differ depending on the individual, and whispers, knocking sounds, and binaural sounds are generally reported as popular content. Therefore, as in the case of using music to induce sleep, ASMR content preferred by each subject or showing a neural response may be different. Therefore, more diverse studies such as research by ASMR content and studies reflecting the subject’s preference are needed.

### 5.2. Treatment Satisfaction Questionaries for Medication

In order to provide a service that makes it easier to fall asleep using auditory stimulation, it is necessary to evaluate the effect of reducing the subjective sleep latency that people actually feel. The papers that evaluated the usefulness of the sleep-inducing effects of auditory stimulation reviewed in this paper were generally studied with well-characterized and carefully selected patient samples using rigorous experimental designs. The objective report that sleep latency was significantly reduced by the sleep diary or polysomnography test organized through these studies is academically significant. However, it is also necessary to personally evaluate whether people who actually judge themselves to be unable to fall asleep easily fall asleep when exposed to auditory stimulation. This is because, although there is a statistically significant improvement in sleep latency, if people do not subjectively feel the effect of the improvement, the quality of the service is lowered. For this reason, it is also necessary to evaluate the user experience related to the sleep-inducing effect of auditory stimulation.

Patient-reported outcomes, in particular, patient satisfaction, are increasingly being recognized as important in determining the efficacy of new therapies, such as digital therapeutics. Patient satisfaction was found to have an effect on patients’ health-related decisions and treatment-related behaviors, which in turn had a significant effect on the success of treatment outcomes [60,61]. For example, the patient’s satisfaction with the medical services they receive has been shown to be highly correlated with successful treatment, compliance with medical care, follow-up to treatment plans, and proper use of services [62,63,64]. Similarly, patient satisfaction with medication has been shown to influence treatment-related behaviors such as the likelihood of continuing to use the medication, using the medication correctly, and following a medication regimen [65,66,67,68]. Atkinson et al. developed a Treatment Satisfaction Questionnaire for Medication [TSQM] tool applicable to various chronic diseases [69]. The items of the tool measure satisfaction with the effect of the currently administered drug, side effects, discomfort, and the degree of impact on daily life. For biopharmaceuticals of the traditional concept manufactured using materials derived from humans or other organisms, such as biologics, genetically modified drugs, cell-cultured drugs, cell therapy products, and gene therapy products, there is some literature examining treatment satisfaction with medication using TSQM. Bagel et al. used TSQM-9 (a simplified version excluding side effects related questions) to investigate patient satisfaction with drug treatment in patients with psoriasis vulgaris. As a result of examining drug treatment satisfaction with topical suspensions of calcipotriene and betamethasone dipropionate, the average TSQM-9 score in the areas of treatment effect, convenience, and overall satisfaction at the end of 8 weeks of treatment was 68–74 out of 100 [70]. Whalley et al. investigated the drug treatment satisfaction of Montelukast in an orally disintegrating tablet formulation for asthma and allergic rhinitis patients using TSQM-9. A total of 89.6% of the participants reported overall satisfaction with the oral disintegrating tablet formulation, and the average scores in the global satisfaction and convenience domains were reported to be 58.9 and 66.7, respectively [71]. Digital therapeutics that have been recently proposed, such as auditory stimulation to induce sleep, are also a kind of digital drug to relieve symptoms that have worsened medically or wellness. In order to evaluate the effect of sleep inducers using auditory stimulation, it is of course necessary to quantify how much sleep was induced as an objective diagnostic index through a sleep diary or polysomnography as in the existing literature. In addition, if the following four user experiences are evaluated through a patient satisfaction survey such as TSQM, it will be more helpful in realizing wellness services by presenting both objective and subjective measures for digital sleep inducers: (1) Whether sleep was induced subjectively through auditory stimulation (Effectives), (2) whether there were side effects such as dizziness due to auditory stimulation (side effects), (3) whether it was easy to listen to sound sources on a digital device before going to sleep (convenience), and (4) overall satisfaction (global satisfaction).

### 5.3. Effect of Auditory Stimulation on Sleep

In this review paper, in order to investigate whether sleep is induced through auditory stimulation, the sleep onset latency among the experimental results of various literature has been summarized. Auditory stimulation not only affects sleep onset latency but also affects total sleep time, sleep efficiency, wake time after sleep onset (WASO), and sleep structure (change in the proportion of each sleep stage in total sleep time). Therefore, it is necessary to conduct a clinical study on what kind of relationship these sleep-related parameters according to auditory stimulation have with each other, and how quality sleep is actually achieved when each parameter is changed. In addition, in the midst of such various influences, it is also required to choose which parameter should be prioritized for R&D for service implementation. The purpose of providing auditory stimulation as a sleep service in a healthcare device is to obtain good quality sleep. Quality sleep is a commonly used term, but it has not yet been well defined. Akerstedt et al. commented that “in fact, there seems to be little systematic knowledge about what constitutes subjectively good sleep and how to measure it” [72], and Buysse et al. define sleep quality as “complex phenomena that are difficult to define and measure objectively” [73]. According to a study conducted by Harvey et al., which investigated the subjective meaning of people’s perceptions of sleep quality, the most common item that people with insomnia and the healthy general public responded to as being related to quality sleep was “Motivation to get up or sleep in the morning”, “Tiredness on waking and throughout the day”, “Sleep onset latency”, and “Awakenings in the night” [74]. Among them, the first two are items corresponding to the waking daytime, and the latter two are the most important factors for a quality sleep subjectively felt at night. Most of the awakening during night sleep is not recognized by the actual sleeper, and even in the case of perceived awakening, it is important to fall asleep quickly. Therefore, if the sleep onset latency is shortened through auditory stimulation and it is possible to fall asleep quickly, it will be useful for users who use this sleep induction service to subjectively feel the service effectiveness.

### 5.4. Sleep Improving Evidence

Dickson and Schubert reviewed the literature on the sleep-improving effects of auditory stimulation and summarized the sleep-improving evidence suggested by researchers in six statements [72]: (1) Relaxation effect: auditory stimulation promotes physiological or psychological relaxation, (2) distraction: music plays a focal role to distract from internal stressful thoughts, (3) synchronization: matching of biological rhythms to the beat of the auditory stimulus source, (4) sound masking: blocking distracting background noise with music, (5) enjoyment: listening to preferred, emotionally relevant, or pleasurable music, and (6) expectations: personal cultural beliefs about music.

Among the studies we investigated, most of the sleep-inducing effects caused by auditory stimulation corresponding to pure noise are due to sound masking. In sound masking, the arousal caused by distracting external background noise can be minimized by continuously reproducing pure colored noise at a relatively greater intensity. A representative study of this evidence was conducted by Stanchina et al. [73]. In this study, sound sources recorded in the intensive care unit, including patient care activities, nurse-patient interactions, periodic alarms from mechanical ventilators, infusion pumps, and mixed background noise, were present during polysomnography. The experiment reported that arousal during sleep increased when exposed to only noise recorded in the intensive care unit but not when exposed to both ICU noise and white noise. Among the studies we investigated is one conducted in a hospital or university dormitory that indicated that pure colored noise masked external noise to induce sleep by minimizing arousal. However, the experiment conducted in the polysomnography laboratory removed almost all external noise, limiting the conclusion that the sleep-inducing effect of pure noise is due to masking.

In the studies that used music as an auditory stimulus for sleep induction, all except one by Iwaki et al. [50] described the music content they used, and most was stable and sedative music with a slow tempo. Johnson et al. did not impose any restrictions on the choice of music, allowing subjects to choose freely [51]. The majority of participants opted for soothing classical music such as Pachelbel’s Canon D or Bach at Bedtime. A study by Lai and Good asked participants to choose one of five types of Western music and one piece of Chinese music with a tempo between 60 and 80 beats per minute without accented beats, percussive characteristics, or syncopation [52]. Music was composed specifically in a study by Bloch of a modal harmonic progression of Am/C played at a tempo of 52 bpm and a moderate volume with no dynamic changes throughout [49]. In a study by Shum et al., participants were able to choose their preferred music among Western classics, Chinese classics, new age, and jazz [53]. All music compositions were smooth and instrumental, with no lyrics and slow music at around 60–80 beats per minute. In the study by Wang et al., the desired music was selected from a music database of various types of music including Chinese instrumental classical music, Western classical music, natural sound music, and classical music without lyrics [54]. All music selected by the subjects was soft and sedative, with stable melodies at a tempo of 60–80 beats per minute. Dubey et al. concluded that 432 Hz music had a significant sedative effect as reflected by increased alpha activity [55]. In the study by Jepersen et al., subjects were able to select the desired sound source among classical, jazz, new age, and ambient music characterized by a slow tempo (50~80 bpm), stable dynamics, and simple structure, and these characteristics were said to be optimal for relaxation [56].

Due to the characteristics of the music used in the studies, it is interpreted that most promoted sleep through physiological and psychological “relaxation” fostered by auditory stimulus. Such effects can be interpreted as “enjoyment”, as has been established in the study by Talbot et al. [74]. They compared the mood states of happy, sad, and neutral music at bedtime for sleep in healthy individuals. Music can induce mood, and both happy and sad music reduce sleep latency in healthy individuals compared to neutral music. The music used in the studies that we investigated is characterized by a slow and stable tempo. Not all of these sound sources are sadness-inducing, although it is likely that sadness-inducing music will have a slow and stable tempo. Therefore, a sad mood can be experienced through relaxation, which can induce sleep. Moreover, this slow-tempo, steady, relaxing music created a “distraction” from stressful thoughts, implying a relationship with relaxation.

In studies using ASMR as an auditory stimulus, the same sleep inducement basis as in music can be applied. In the studies by Williamson and Umbas, natural sounds such as waterfall or rain were used as sound sources, which can mask surrounding noise similarly to white noise [44,46]. In Williamson, since the experiment focused on night sleep of patients in an intensive care unit, it is judged that sound masking caused by natural sounds helped to induce sleep under conditions similar to that found in the study by Stanchina et al. [44,73]. Since the study of Umbas et al. was conducted in the dormitory, it is thought that masking by sounds of nature helped to induce sleep in an environment containing various living noises [46]. For the ASMR sound sources we found on YouTube, it is judged that a sound masking effect was created because the frequency characteristic with time has the characteristics of intermittent white or pink noise. In addition, research shows that listening to the sounds of nature can affect the body to calm and relax [75]. Therefore, the sounds of nature, including the sound of rain, create a calm atmosphere for meditation and, as a result, induce sleep by relaxation. Hardian et al. used ASMR sound sources such as whispering sounds and rubbing objects while role playing [45]. This kind of ASMR can be defined as a combination of pleasure and relaxation preceded by a specific tingling sensation [43]. The subjective experience of tingling caused by ASMR is often accompanied by feelings of stillness and relaxation. Therefore, it can be interpreted that ASMR induces sleep by relaxation in addition to sound masking.

The sleep-inducing effect of auditory stimulation can also be based on “expectation”. It is believed that auditory stimulation itself is not an active process for inducing sleep, but rather acts as a kind of placebo. In other words, just believing that auditory stimulation helps in falling asleep can be enough to produce an improvement. It is a common finding that a placebo has a significant effect on measured health status. Drugs such as Zolpidem or Flurazepam effectively improve sleep delay time compared to placebo, but there are reports of placebo improving sleep delay time [76,77]. In a recent meta-analysis, McCall et al. concluded that the literature (a total of 213 insomnia patients across 5 articles) demonstrated a significant decrease in subjective sleep latency of 13.1 ± 2.0 min for the placebo group [78]. In addition, Perlis and colleagues reported that the effect of placebo appeared to be potent and long lasting [79]. Auditory stimulation can be a kind of placebo. If we believe that exposure to auditory stimuli such as colored noise, music, or ASMR helps us fall asleep, it will have the same effect as a placebo in reducing sleep latency, and long-term exposure to auditory stimuli strongly affects sleep latency.

### 5.5. Potential for Digital Therapeutic Applications

Digital therapeutics, which use digital technology as therapeutic drugs, have been recognized for their effectiveness and are expected to spread across the global market. Research and development of digital therapeutics for insomnia are progressing actively, with efficacy verification through clinical research and further FDA approval. Big Health’s Sleepio has accumulated significant clinical research results and has established a firm position in the digital therapeutics market [80,81,82,83]. Although notable clinical research results have been secured, they are from the perspective of public health rather than others such as US FDA approval. Another representative case is SOMRYST of Pear Therapeutics, which reported an improvement of secondary indicators through clinical research, for instance, an improvement not only in symptoms of insomnia, but also depression [84,85,86]. This digital treatment for insomnia has been approved by the US FDA. In addition, there are digital therapeutics introduced by BetterNight and Mindware Consulting, all of which can be seen as an implementation of cognitive behavioral therapy as a smartphone app. All of these are considered meaningful treatments whose effectiveness has been verified through clinical studies. However, the auditory stimulation-based sleep induction method has the advantage of being popularized as a sleep inducer for the general public rather than as a medicine prescribed by medical staff. Until now, digital therapeutics for insomnia have been limited to implementation of cognitive behavioral therapy as software for a smartphone app. No matter the content and treatment techniques, if the user experience and convenience do not support them, the user’s subjective satisfaction is halved, and compliance is reduced. As cognitive behavioral therapy requires active intervention and effort from the users, its convenient and continuous use by the general public before diagnosis of insomnia is limited. For digital therapeutics, continuous use of therapeutic agents by stable adherence is a key factor in achieving therapeutic effects. Therefore, as the first step for treating insomnia, an auditory stimulation method has the added strength of a sleep-inducing digital therapy that many people can try without a prescription from medical staff. In addition, the auditory stimulus-based method is easy to integrate with various Internet of Things (IoT) devices. Although various attempts and developments have been made to measure the state of sleep through wearable devices, etc., there is doubt about the usefulness and value of simply measuring the state of sleep accurately [87,88]. The advancement of diagnostic technology must be followed by therapeutic application. With this concept, the integrated application of various IoT technologies is required, and the sleep-inducing effect through auditory stimulation will be a therapeutic application that can satisfy these needs.

## 6. Conclusions

Initial digital treatment can be used by the general public to aid in sleeping before being diagnosed with insomnia and proceeding with drug or non-drug treatment prescribed by a doctor. The most important factor in the expansion of digital therapeutics is objective clinical data that can be used to judge safety and efficacy. To provide a service that can induce sleep through auditory stimulation, more clinical research on the sleep-inducing effect of auditory stimulation is needed. Most of the related studies have been based on sleep diaries. Therefore, clinical studies based on polysomnography of subjects with various conditions are needed to determine meaningful clinical effectiveness. In particular, research on methods using ASMR is almost nonexistent, and more research is necessary.

## Figures and Tables

**Table 1:** Representative auditory stimulation-based sleep induction devices and apps on the market.

Product Category	Product Name	Sound Material	Additional Functions	Photo
Sound machine	Sound + Sleep Mini	12 audio programs, each with multiple options for environment and complexity, for a total of 48 unique audio settings (rainfall, flowing water, ocean, city, white/pink/brown noise, etc.)	Built-in microphone to listen to your environment and dynamically adjust the volume based on ambient noise levels	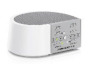
Sound machine	Lectrofan	12 unique digital sounds to mask noises and a choice from 10 electric fan sounds and 10 variations of pure white noise (including pink and brown noise variations)	Soft-glow reading light without eye-straining blue hues	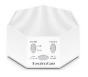
Sound machine	Hatch Restore	Library of soothing sounds (white noise, ocean, rain, water, wind, birds, dryer, heartbeat, etc)	Sunrise alarm, smart light, meditation app, and an alarm clock	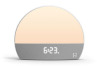
Sound machine	WAVE™	6 soothing sounds (white noise, fan, ocean, rain, stream, and summer night)	USB port with 2A output to charge USB-powered device	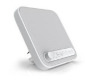
Sound machine	Letsfit T126L	14 natural sounds (white noise, ocean wave, crickets, fan noise, clothes dryer, birds, thunderstorm, water stream, nursery rhyme)	Warm night-light	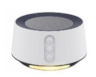
Wearable	Sleepphones	17 audio tracks featuring binaural beat technology (colored noise, waterfall, stream, waves, etc.)	For listening to music and TV in bed at night while your partner sleeps	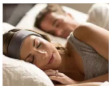
App	Sleepmaker Rain1	21 rain sounds (rainfall in forests, rain against window with wind, rain onto porch roof, torrential downpour, etc.)	Programmable Sleep Timer	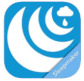
App	Sleepa	Sleepa Sound Library (rain, forest, creek, wind, fire, city sound, colored noise, binaural beats that replicate brainwaves)	Mix, match, and save over 120 M combinations of soundsProgrammable Sleep Timer	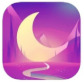

**Table 2:** A study on sleep induction using pure colored noise as an auditory stimulus.

First Author, Year, Country	Study Design	Participants	N, Mean Age ± SD (or Range)	Noise (Method)	Intervention Duration	Control Group	Sleep Measure(s)	Sleep Outcomes
Spencer (1990) England	Randomized controlled trial	Healthy neonates	40, (2–7 days old)	White noise (speaker)	Four and a half minutes	Not exposed to white noise	Observed by a single investigator	80% of babies fell asleep when they were exposed to the white noise compared with 25% who fell asleep in the control group.
Forquer&Johnson (2007) USA	Crossover	Healthy college students	4, 19	White noise (speaker)	Continuously from bedtime to waking for 7 nights	None	Sleep diary, PSQI, sleep hygiene test	Decrease in both sleep latency and night wakings during treatment
Rosalez (2020)	Crossover	ADHD Children	3 (9–11)	White noise (speaker)	Continuous at night	Not exposed to white noise	Sleep diary	Decreased bedtime sleep latency and spontaneous night wakings at home
Ebben (2021) USA	Crossover	Adults with insomnia	10, 58 (39–74)	White noise (speaker)	2nd week of 3 weeks,	None	Daily sleep diary and actigraphy	Reduced WASO and sleep latency, reduced the number of awakenings during the night, improved sleep efficiency
Cho (2021) Korea	Randomized controlled trial	Adult inpatient	61	White noise (earphone)	1 h per night for 3 days	Wear earplugs	VSH, actigraphy	Positive effects on subjective sleep quality, improved sleep time and sleep efficiency
Messineo (2017) USA	Crossover	Healthy adults	18 (20–65)	Broadband (speaker)	1 of 2 weeks	None	PSG, PSQI, VAS, SSS	Reduced sleep onset latency, improved subjective sleep quality
Kawada (1993) Japan	Crossover	Healthy young subjects	4, 19.8 (19–21)	Pink noise (speaker)	1 of 2 days	None	PSG	Reduced sleep latency
Garcia-Molina (2020) USA	Crossover	Healthy subjects	7, 33.6 ± 8.7	Pink noise (headband)	4 of 5 sleep sessions	None	PSG	Reduced slap latency

**Table 3:** ASMR for sleep inducement with the highest number of views uploaded in 2021 (accessed on: 7 February 2022).

Num	Sound	URL	Total Views
(a)	The sound of making glossy slime with a piping bag	https://youtu.be/NaHEoL1Zs-E	3,113,664
(b)	A voice whispering softly during a hair shop role play	https://youtu.be/yWIXozR85c0	1,694,665
(c)	The sound of an oil massage and the whispering sound overlapped	https://youtu.be/j71CE1dpwmw	681,237
(d)	The sound of raindrops falling from the eaves and raindrops pouring	https://youtu.be/Ol5CxwbC5ag	746,214
(e)	Steel wool sponges, the sound of rubbing various brushes, dolls, paper packaging materials, toys, etc.	https://youtu.be/We02eW1950M	500,158

**Table 4:** Research studies on sleep induction using ASMR as auditory stimulation.

First Author, Year, Country	Study Design	Participant	N, Mean Age ± SD (or Range)	Noise (Method)	Intervention Duration	Control Group	Sleep Measure(s)	Sleep Outcomes
Williamson (1992) USA	Randomized controlled trial	First-time CABG patients	60	Soothing nature sounds such as rain, waves, or waterfalls (speaker)	Three consecutive nights posttransfer from the ICU	Not exposed to ocean sound	Richards–Campbell Sleep Questionnaire, a visual analog scale	Falling asleep faster and maintaining sleep
Hardian (2020) Indonesia	Randomized controlled trial	Students at medical college	30, 18.4 ± 0.72, 17~20	Haircut, cooking, whispering, rubbing, delicate hand movements (earphone)	14 days, 20 min from 9:00 pm	Not exposed to ASMR	PSQI	Improved subjective sleep quality
Umbas (2021) Indonesia	Crossover	High school students	12 (16–18)	Rain sound (speaker)	Whole night for 30 days	None	PSQI	Reducing sleep onset latency Triggering deeper sleep

**Table 5:** A study on sleep induction using music as an auditory stimulus.

First Author, Year, Country	Study Design	Participant	N, Mean Age ± SD (or Range)	Noise (Method)	Intervention Duration	Control Group	Sleep Measure(s)	Sleep Outcomes
Iwaki (2003) Japan	Crossover	Healthy university students	20 (20–28)	Preferred familiar music (speaker)	1 week before nap	No music played	PSG, KSS	Reduced sleep latency in natural sleep
Johnson (2003) USA	Crossover	Older subjects with insomnia	52 (80.5, 71–87)	Participant selected music (NA)	turn the music on as soon as they got into bed for 10 nights	No music played	Stanford Sleepiness Scale Sleep log	Significant increase in level of sleepiness at bedtime Significant decrease in time to sleep onset and number of nighttime awakenings
Lai (2006) USA	Randomized controlled trial	Subjects with difficulty in sleeping	60 (60~83)	five types of Western and one of Chinese music (NA)	45 min at bedtime for 3 weeks	No music played	PSQI, ESC	Better sleep quality, better perceived sleep quality, longer sleep duration, greater sleep efficiency, shorter sleep latency, less sleep disturbance, and less daytime dysfunction
Bloch (2010) Israel	Crossover	Subjects with schizophrenia	24, 45.67 ± 9.6 (18~70)	Relaxation music composed specifically for the study (speaker)	40 min at bedtime, 7 of 14 days	No intervention	Actigraphy, Subjective sleep questionnaires	Reduced sleep latency, improved sleep efficiency, reduction in the number of reported complaints of subjective insomnia
Shum (2014) Singapore	Randomized controlled trial	Community-dwelling older adults	60, 55 years or above	Soft, instrumental slow sedative music without lyrics (earphone)	40 min per night for 6 weeks	Not listen to sedative music, but they were not restricted from listening to radio or TV	PSQI	Improving sleep quality
Wang (2016) China	Randomized controlled trial	Older with poor sleep quality	64, 69.38 ± 5.46	Preferred music (earphone)	30–45 min per night for 3 months	No intervention	PSQI, QoS	Improving sleep latency, sleep efficiency, and daytime dysfunction
Dubey (2019) India	Crossover	Healthy male volunteer with a history of delayed sleep latency	15 (18~40)	432 Hz music (NA)	15 to 20 min before nap	No intervention	PSG	Decrease in the mean sleep latency, increase in the energy of alpha waves at the sleep onset.
Jespersen (2019) Denmark	Randomized controlled trial	Subjects with insomnia	57 (18 and 65)	Classical, jazz, new age, and ambient music (NA)	Minimum 30-min at bedtime for 3 weeks	No intervention	PSG, PSQI, pQoL	Positive impact on sleep perception and quality of life, reduced sleep onset latency

## References

[1] Naomi B., Thomas R., Leon R., Andreski P. (1996). Sleep Disturbance and Psychiatric Disorders: A Longitudinal Epidemiological Study of Young Adults. Biol. Psychiatry.

[2] Lichstein K.L., Durrence H.H., Taylor D.J., Bush A.J., Riedel B.W. (2003). Quantitative criteria for insomnia. Behav. Res. Ther..

[3] Hughes R.J., Sack R.L., Lewy A.J. (1998). The role of melatonin and circadian phase in age-related sleep-maintenance insomnia: Assessment in a clinical trial of melatonin replacement. Sleep.

[4] Roth T., Walsh J.K., Krystal A., Wessel T., Roehrs T.A. (2005). An evaluation of the efficacy and safety of eszopiclone over 12 months in patients with chronic primary insomnia. Sleep Med..

[5] Espie C.A., Brooks D.N., Lindsay W.R. (1989). An evaluation of tailored psychological treatment of insomnia. J. Behav. Ther. Exp. Psychiatry.

[6] Wu R., Bao J., Zhang C., Deng J., Long C. (2006). Comparison of sleep condition and sleep-related psychological activity after cognitive-behavior and pharmacological therapy for chronic insomnia. Psychother. Psychosom..

[7] American Academy of Sleep Medicine (2014). International Classification of Sleep Disorders.

[8] American Psychiatry Association (2013). Diagnostic and Statistical Manual of Mental Disorders.

[9] Walsh J.K., Coulouvrat C., Hajak G., Lakoma M.D., Petukhova M., Roth T., Sampson N.A., Shahly V., Shillington A., Stephenson J.J. (2011). Nighttime insomnia symptoms and perceived health in the America Insomnia Survey (AIS). Sleep.

[10] Roth T., Coulouvrat C., Hajak G., Lakoma M.D., Sampson N.A., Shahly V., Shillington A.C., Stephenson J.J., Walsh J.K., Kessler R.C. (2011). Prevalence and perceived health associated with insomnia based on DSM-IV-TR; International Statistical Classification of Diseases and Related Health Problems, Tenth Revision; and Research Diagnostic Criteria/International Classification of Sleep Disorders, Second Edition criteria: Results from the America Insomnia Survey. Biol. Psychiatry.

[11] Morin C.M., LeBlanc M., Daley M., Gregoire J.P., Merette C. (2006). Epidemiology of insomnia: Prevalence, self-help treatments, consultations, and determinants of help-seeking behaviors. Sleep Med..

[12] Ohayon M.M. (2002). Epidemiology of insomnia: What we know and what we still need to learn. Sleep Med. Rev..

[13] Roth T. (2007). Insomnia: Definition, prevalence, etiology, and consequences. J. Clin. Sleep Med..

[14] Ohayon M.M., Hong S.C. (2002). Prevalence of insomnia and associated factors in South Korea. J. Psychosom. Res..

[15] Cho Y.W., Shin W.C., Yun C.H., Hong S.B., Kim J., Earley C.J. (2009). Epidemiology of insomnia in korean adults: Prevalence and associated factors. J. Clin. Neurol..

[16] Chung S., Cho S.W., Jo M.W., Youn S., Lee J., Sim C.S. (2020). The Prevalence and Incidence of Insomnia in Korea during 2005 to 2013. Psychiatry Investig..

[17] Wang C., Song W., Hu X., Yan S., Zhang X., Wang X., Chen W. (2021). Depressive, anxiety, and insomnia symptoms between population in quarantine and general population during the COVID-19 pandemic: A case-controlled study. BMC Psychiatry.

[18] Voitsidis P., Gliatas I., Bairachtari V., Papadopoulou K., Papageorgiou G., Parlapani E., Syngelakis M., Holeva V., Diakogiannis I. (2020). Insomnia during the COVID-19 pandemic in a Greek population. Psychiatry Res..

[19] Şahin M.K., Aker S., Şahin G., Karabekiroğlu A. (2020). Prevalence of Depression, Anxiety, Distress and Insomnia and Related Factors in Healthcare Workers During COVID-19 Pandemic in Turkey. J. Community Health.

[20] Li Y., Qin Q., Sun Q., Sanford L.D., Vgontzas A.N., Tang X. (2020). Insomnia and psychological reactions during the COVID-19 outbreak in China. J. Clin. Sleep Med..

[21] Danzig R., Wang M., Shah A., Trotti L.M. (2020). The wrist is not the brain: Estimation of sleep by clinical and consumer wearable actigraphy devices is impacted by multiple patient- and device-specific factors. J. Sleep Res..

[22] Edinger J.D., Wohlgemuth W.K., Radtke R.A., Marsh G.R., Quillian R.E. (2001). Cognitive behavioral therapy for treatment of chronic primary insomnia: A randomized controlled trial. JAMA.

[23] Morin C.M., Colecchi C., Stone J., Sood R., Brink D. (1999). Behavioral and pharmacological therapies for late-life insomnia: A randomized controlled trial. JAMA.

[24] Luik A.I., Kyle S.D., Espie C.A. (2017). Digital cognitive behavioral therapy (dCBT) for insomnia: A state-of-the-science review. Curr. Sleep Med. Rep..

[25] Seyffert M., Lagisetty P., Landgraf J., Chopra V., Pfeiffer P.N., Conte M.L., Rogers M.A. (2016). Internet-delivered cognitive behavioral therapy to treat insomnia: A systematic review and meta-analysis. PLoS ONE.

[26] Koffel E., Bramoweth A.D., Ulmer C.S. (2018). Increasing access to and utilization of cognitive behavioral therapy for insomnia (CBT-I): A narrative review. J. Gen. Intern. Med..

[27] Coulson N.S., Smedley R., Bostock S., Kyle S.D., Gollancz R., Luik A.I., Hames P., Espie C.A. (2016). The Pros and Cons of Getting Engaged in an Online Social Community Embedded within Digital Cognitive Behavioral Therapy for Insomnia: Survey among Users. J. Med. Internet Res..

[28] Thomas K.A., Martin P.A. (2000). NICU sound environment and the potential problems for caregivers. J. Perinatol..

[29] Tomei F., Papaleo B., Baccolo T.P., Persechino B., Spano G., Rosati M.V. (1994). Noise and gastric secretion. Am. J. Ind. Med..

[30] Falk S.A., Woods N. (1973). Hospital noise: Levels and potential health hazards. N. Engl. J. Med..

[31] Nurminen T. (1995). Female noise exposure, shift work and reproduction. J. Occup. Environ. Med..

[32] Trahan T., Durrant S.J., Müllensiefen D., Williamson V.J. (2018). The music that helps people sleep and the reasons they believe it works: A mixed methods analysis of online survey reports. PLoS ONE.

[33] Huang C.-Y., Chang E.-T., Lai H.-L. (2018). Use of integrative medicine approaches for treating adults with sleep disturbances. Appl. Nurs. Res..

[34] Spencer J.A., Moran D.J., Lee A., Talbert D. (1990). White noise and sleep induction. Arch. Dis. Child..

[35] Forquer L.M., Johnson C.M. (2007). Continuous White Noise to Reduce Sleep Latency and Night Wakings in College Students. Sleep Hypn..

[36] Ebben M.R., Yan P., Krieger A.C. (2021). The effects of white noise on sleep and duration in individuals living in a high noise environment in New York City. Sleep Med..

[37] Cho M.E., Hwang S.K. (2021). The Effect of White Noise on Sleep in Hospitalized Patients: A Randomized Controlled Trial. Korean J. Adult Nurs..

[38] Messineo L., Taranto-Montemurro L., Sands S.A., Oliveira Marques M.D., Azabarzin A., Wellman D.A. (2017). Broadband Sound Administration Improves Sleep Onset Latency in Healthy Subjects in a Model of Transient Insomnia. Front. Neurol..

[39] Papalambros N.A., Santostasi G., Malkani R.G., Braun R., Weintraub S., Paller K.A., Zee P.C. (2017). Acoustic Enhancement of Sleep Slow Oscillations and Concomitant Memory Improvement in Older Adults. Front. Hum. Neurosci..

[40] Zhou J., Liu D., Li X., Ma J., Zhang J., Fang J. (2012). Pink noise: Effect on complexity synchronization of brain activity and sleep consolidation. J. Theor..

[41] Kawada T., Suzuki S. (1993). Sleep induction effects of steady 60 dB (A) pink noise. Ind. Health.

[42] Garcia-Molina G., Kalyan B., Aquino A. (2020). Closed-Loop Electroencephalogram-Based Modulated Pink Noise to Facilitate Falling Asleep. Sleep.

[43] Poerio G.L., Blakey E., Hostler T.J., Veltri T. (2018). More than a feeling: Autonomous sensory meridian response (ASMR) is characterized by reliable changes in affect and physiology. PLoS ONE.

[44] Williamson J.W. (1992). The effects of ocean sounds on sleep after coronary artery bypass graft surgery. Am. J. Crit. Care.

[45] Hardian H., Febriani S.S., Sumekar T.A., Muniroh M., Indraswari D.A., Purwoko Y., Ambarwati E. (2020). Improvement of Sleep Quality by Autonomous Sensory Meridian Response (ASMR) Stimulation Among Medical Students. Mal. J. Med. Health Sci..

[46] Umbas J.C.G., Bintang A.K., Aulina S., Bahar A., Akbar M. (2021). The effect of white noise on high school students’ sleep quality at Unit B of Rajawali Girls Dormitory Makassar. Med. Clínica Práctica.

[47] Lee T., Moon S.E., Baek J., Lee J.S., Kim S. (2019). Music for Sleep and Wake-Up: An Empirical Study. IEEE Access.

[48] Loewy J. (2020). Music Therapy as a Potential Intervention for Sleep Improvement. Nat. Sci. Sleep.

[49] Bloch B., Reshef A., Vadas L., Haliba Y., Ziv N., Kremer I., Haimov I. (2010). The effects of music relaxation on sleep quality and emotional measures in people living with schizophrenia. J. Music Ther..

[50] Iwaki T., Tanaka H., Hori T. (2003). The Effects of Preferred Familiar Music on Falling Asleep. J. Music Ther..

[51] Johnson J.E. (2003). The use of music to promote sleep in older women. J. Community Health Nurs..

[52] Lai H.L., Good M. (2005). Music improves sleep quality in older adults. J. Adv. Nurs..

[53] Shum A., Taylor B.J., Thayala J., Chan M.F. (2013). The effects of sedative music on sleep quality of older community-dwelling adults in Singapore. Complement. Ther. Med..

[54] Wang Q., Chair S.Y., Wong E.M.L., Li X. (2016). The Effects of Music Intervention on Sleep Quality in Community-Dwelling Elderly. J. Altern. Complement. Med..

[55] Dubey P., Kumar Y., Singh R., Jha K., Kumar R. (2019). Effect of music of specific frequency upon the sleep architecture and electroencephalographic pattern of individuals with delayed sleep latency: A daytime nap study. J. Fam. Med. Prim. Care.

[56] Jespersen K.V., Otto M., Kringelbach M., van Someren E., Vuust P. (2019). A randomized controlled trial of bedtime music for insomnia disorder. J. Sleep Res..

[57] McCall C., McCall W.V. (2012). Comparison of actigraphy with polysomnography and sleep logs in depressed insomniacs. J. Sleep Res..

[58] Kaplan K.A., Talbot L.S., Gruber J., Harvey A.G. (2012). Evaluating sleep in bipolar disorder: Comparison between actigraphy, polysomnography, and sleep diary. Bipolar Disord..

[59] Matthews K.A., Patel S.R., Pantesco E.J., Buysse D.J., Kamarck T.W., Lee L., Hall M.H. (2018). Similarities and differences in estimates of sleep duration by polysomnography, actigraphy, diary, and selfreported habitual sleep in a community sample. Sleep Health.

[60] Brody D., Miller S., Lerman C., Smith D., Caputo G. (1989). Patient perception of involvement in medical care: Relationship to illness attitudes and outcomes. J. Gen. Intern. Med..

[61] Taylor T.R. (2000). Understanding the choices that patients make. J. Am. Board Fam. Pract..

[62] Albrecht G., Hoogstraten J. (1998). Satisfaction as a determinant of compliance. Community Dent. Oral Epidemiol..

[63] McCracken L.M., Klock A., Mingay D.J., Asbury J.K., Sinclair D.M. (1997). Assessment of satisfaction with treatment for chronic pain. J. Pain Symptom Manag..

[64] Weaver M., Patrick D.L., Markson L.E., Martin D., Frederic I., Berger M. (1997). Issues in the measurement of satisfaction with treatment. Am. J. Manag. Care.

[65] Zhang Z., Gerstein D.R., Friedmann P.D. (2008). Patient satisfaction and sustained outcomes of drug abuse treatment. J. Health Psychol..

[66] Awad A.G., Voruganti L.N.P. (1999). Quality of life and new antipsychotics in schizophrenia. Are patients better off?. Int. J. Soc. Psychiatry.

[67] Diamond R. (1985). Drugs and the quality of life: The patient’s point of view. J. Clin. Psychiatry.

[68] Testa M.A., Simonson D.C. (2007). Satisfaction and quality of life with premeal inhaled versus injected insulin in adolescents and adults with type 1 diabetes. Diabetes Care.

[69] Atkinson M.J., Sinha A., Hass S.L., Colman S.S., Kumar R.N., Brod M., Rowland C.R. (2004). Validation of a general measure of treatment satisfaction, the Treatment Satisfaction Questionnaire for Medication (TSQM), using a national panel study of chronic disease. Health Qual. Life Outcomes.

[70] Bagel J., Levi E., Tyring S., Knuckles M.L. (2014). Real-life treatment profile of calcipotriene and betamethasone dipropionate topical suspension in patients with psoriasis vulgaris. J. Drugs Dermatol..

[71] Whalley D., Petigara T., Rasouliyan L., Tobe K., Tunceli K. (2017). Early patient experiences with montelukast orally disintegrating tablets in Japan: A cross-sectional survey of treatment satisfaction in patients with asthma and/or allergic rhinitis. Curr. Med. Res. Opin..

[72] Dickson G.T., Schubert E. (2019). How does music aid sleep? Literature review. Sleep Med..

[73] Stanchina M.L., Abu-Hijleh M., Chaudhry B.K., Carlisle C.C., Millman R.P. (2005). The influence of white noise on sleep in subjects exposed to ICU noise. Sleep Med..

[74] Talbot L.S., Hairston I.S., Eidelman P., Gruber J., Harvey A.G. (2009). The effect of mood on sleep onset latency and REM sleep in interepisode bipolar disorder. J. Abnorm. Psychol..

[75] Yu B., An P., Hendriks S., Zhang N., Feijs L., Li M., Hu J. (2021). ViBreathe: Heart Rate Variability Enhanced Respiration Training for Workaday Stress Management via an Eyes-Free Tangible Interface. Int. J. Hum. -Comput. Int..

[76] Roth T., Soubrane C., Titeux L., Walsh J.K. (2006). Efficacy and safety of zolpidem-MR: A double-blind, placebo-controlled study in adults with primary insomnia. Sleep Med..

[77] Scharf M.B., Roth P.B., Dominguez R.A., Ware J.C. (1990). Estazolam and Flurazepam: A Multicenter, Placebo-Controlled Comparative Study in Outpatients with Insomnia. J. Clin. Pharmacol..

[78] McCall W.V., D’Agostino R., Dunn A. (2003). A meta-analysis of sleep changes associated with placebo in hypnotic clinical trials. Sleep Med..

[79] Perlis M.L., McCall W.V., Jungquist C.R., Pigeon W.R., Matteson S.E. (2005). Placebo effects in primary insomnia. Sleep Med. Rev..

[80] Elison S., Ward J., Williams C., Espie C., Davies G., Dugdale S., Ragan K., Chisnall L., Lidbetter N., Smith K. (2017). Feasibility of a UK community-based, eTherapy mental health service in Greater Manchester: Repeated-measures and between-groups study of ‘Living Life to the Full Interactive’, ‘Sleepio’ and ‘Breaking Free Online’ at ‘Self Help Services’. BMJ Open.

[81] Cheng P., Kalmbach D.A., Tallent G., Joseph C.L., Espie C.A., Drake C.L. (2019). Depression prevention via digital cognitive behavioral therapy for insomnia: A randomized controlled trial. Sleep.

[82] Espie C.A., Emsley R., Kyle S.D., Gordon C., Drake C.L., Siriwardena A.N., Cape J., Ong J.C., Sheaves B., Foster R. (2019). Effect of digital cognitive behavioral therapy for insomnia on health, psychological well-being, and sleep-related quality of life: A randomized clinical Trial. JAMA Psychiatry.

[83] Felder J.N., Epel E.S., Neuhaus J., Krystal A.D., Prather A.A. (2020). Efficacy of digital cognitive behavioral therapy for the treatment of insomnia symptoms among pregnant women: A randomized clinical trial. JAMA Psychiatry.

[84] Ritterband L.M., Thorndike F.P., Ingersoll K.S., Lord H.R., Gonder-Frederick L., Frederick C., Quigg M.S., Cohn W.F., Morin C.M. (2017). Effect of a web-based cognitive behavior therapy for insomnia intervention with 1-year follow-up: A randomized clinical trial. JAMA Psychiatry.

[85] Morin C.M. (2020). Profile of somryst prescription digital therapeutic for chronic insomnia: Overview of safety and efficacy. Expert Rev. Med. Devices.

[86] Vedaa Ø., Kallestad H., Scott J., Smith O.R., Pallesen S., Morken G., Langsrud K., Gehrman P., Thorndike F.P., Ritterband L.M. (2020). Effects of digital cognitive behavioural therapy for insomnia on insomnia severity: A large-scale randomised controlled trial. Lancet Digit. Health.

[87] Lee H.A., Lee H.J., Moon J.H., Lee T., Kim M.G., In H., Cho C.H., Kim L. (2017). Comparison of wearable activity tracker with actigraphy for sleep evaluation and circadian rest-activity rhythm measurement in healthy young adults. Psychiatry Investig..

[88] Baek H.J., Cho J. (2019). Novel heart rate variability index for wrist-worn wearable devices subject to motion artifacts that complicate measurement of the continuous pulse interval. Physiol. Meas..

